# Glaucomatous Optic Neuropathy: The Dark Side of the Moon

**DOI:** 10.18502/jovr.v16i2.9076

**Published:** 2021-04-29

**Authors:** Shahin Yazdani

**Affiliations:** ^1^Ocular Tissue Engineering Research Center, Shahid Beheshti University of Medical Sciences, Tehran, Iran; ^2^Ophthalmic Research Center, Research Institute for Ophthalmology and Vision Research, Shahid Beheshti University of Medical Sciences, Tehran, Iran

Glaucoma is an enigmatic eye condition and serves as a perfect example for an ocular disease with complex pathophysiology, multifactorial nature, and close interplay with systemic factors. Glaucomatous optic neuropathy (GON) is also probably the most important and prevalent eye condition linked to events within the central nervous system (CNS). None of these is an overstatement when one recalls that the optic nerve head, the site where GON is diagnosed and monitored, is only one small sector of the second cranial nerve, otherwise known as the optic nerve. The major portion of the optic nerve courses in the CNS and acquires physiologic features of neurons residing in the CNS, for example myelination of fibers, just posterior to the lamina cribrosa (LC). Another important fact is that the optic nerve is composed of bundled axons of retinal ganglion cells (RGCs) which have the privilege of “dual citizenship” resting partly in a peripheral organ and partly within the CNS, the boundary being at the LC.

The past decades have witnessed a surge of interest and a wealth of information on previously unknown and less explored aspects of glaucoma. These include the role of systemic factors such as vascular dysregulation, nocturnal systemic hypotension, positional intraocular pressure (IOP) elevation, and episodes of hypoxia due to sleep apnea.^[1,2,3,4,5]^ It has also become more evident that the damage associated with GON is not limited to the optic nerve and secondary/concomitant damage is present at higher centers within the CNS including the lateral geniculate body and primary visual cortex.^[6,7]^ These observations are in favor of the notion that GON represents a neurodegenerative process beyond the eye.

It has become increasingly known that compartments (and pressures within) adjacent to the optic nerve other than the intraocular compartment and IOP have an important role in maintaining its homeostasis.^[8]^ The retrolaminar portion of the optic nerve has attracted much attention and the most important compartment in this region seems to be the subarachnoid space bathed in cerebrospinal fluid (CSF) which is connected to the subarachnoid space of the brain.^[9,10]^

One particular region of interest is the LC, where IOP and CSF pressure (CSFP) meet, exerting pressure on either side of the lamina, thus creating a pressure gradient. The LC has long been considered as a major site of insult to RGCs triggering cell loss and apoptosis. Various in vivo observations using enhanced depth imaging optical coherence tomography (EDI-OCT) and histological studies in animal and human eyes have demonstrated LC changes including displacement, compaction and bowing associated with GON.^[11,12]^ One simplified scheme used to conceptualize glaucomatous damage is to focus on mechanical factors at this region and ignore other physiologic alterations such as impaired nutrition and reduced clearance of neurotoxins. With this simplified mechanical scheme, one can consider IOP and CSFP as two opposing forces which not only affect LC position but also exert stress and strain on this important structure and the delicate retinal nerve fibers protected within the laminar pores. If the normal physiologic balance between these two pressure components is disturbed, the lamina becomes compressed and displaced either posteriorly, as in the case of GON, or anteriorly as in intracranial hypertension.

Having emphasized the importance of the pressure balance at the region of the LC, one cannot neglect the importance of intrinsic LC characteristics such as laminar area, thickness, rigidity/elasticity, compliance, deformability, and pore size. It has been shown that LC thickness may vary in different forms of glaucoma^[13,14,15]^ and might arguably be associated with central corneal thickness, which may serve as a more accessible surrogate biomarker.^[16]^

In this issue of JOVR, Cruz et al^[17]^ report trans-LC pressure gradient (TLCPG) in addition to ocular perfusion pressure (OPP) in a cohort of subjects with definite or suspicious open angle glaucoma stratified by optic disc size. Although this group of investigators found no significant difference in OPP, they reported significantly larger TLCPG in eyes with larger discs. This may reflect higher susceptibility of larger optic nerves to glaucomatous damage. This observation follows basic physical rules such as Laplace's law,^[18]^ which explains that strain in a pressurized hollow structure is directly correlated with its diameter and inversely correlated with its thickness.^[19]^ Although there are limitations to applying purely physical rules to live tissue, the calculated TLCPG reported by the authors seems to be a fairly reasonable reflection of the actual physiologic reality.

The authors are to be commended for their study, but at the same time we need to consider certain limitations of their methodology, partly addressed by the authors. First, CSFP was not actually measured but rather derived mathematically using proxy parameters such as BMI, diastolic blood pressure, and age. However, one may argue that even with lumbar puncture and obtaining CSFP values, the readings may not accurately reflect actual CSFPs at the LC. Second, it is not clear whether major determinants of TLCPG, that is, IOP and CSFP, were comparable among the study groups and that it would be safe to conclude that the observed difference in TLCPG among the study groups was only due to different optic nerve size. Last, it would have been interesting if LC thickness had also been evaluated by EDI–OCT and its measurements had been taken into account.

The study by Cruz et al^[17]^ helps add to the literature much needed data which the ophthalmology community and glaucoma specialists in particular have been missing: the myriad of complex and less recognized factors intertwined in the pathophysiology of GON lurking in less accessible regions of the optic nerve, or “the dark side of the moon.”
